# ASO Author Reflections: The Financial Impact of Delayed and Forgone Care Among Patients with Breast Cancer

**DOI:** 10.1245/s10434-025-16969-8

**Published:** 2025-02-11

**Authors:** Kriyana P. Reddy, Oluwadamilola M. Fayanju

**Affiliations:** 1 Division of Breast Surgery, Department of Surgery, Perelman School of Medicine, The University of Pennsylvania, Philadelphia, PA USA; 2 Rena Rowan Breast Center, Abramson Cancer Center, The University of Pennsylvania, Philadelphia, PA USA; 3 Leonard Davis Institute of Health Economics, The University of Pennsylvania, Philadelphia, PA USA; 4 Penn Center for Cancer Care Innovation, Abramson Cancer Center, The University of Pennsylvania, Philadelphia, PA USA

## Past

Breast cancer has the highest treatment costs of any cancer in the USA, and these costs are projected to rise.^1^ While treatment costs, resource utilization, and outcomes are known to vary by stage at diagnosis, the relationship between delayed and forgone (D/F) care and healthcare resource use and expenditures remains unclear.^2,3^ Understanding and quantifying this relationship is crucial for identifying sources of increased financial burdens on patients with breast cancer who experience delayed care and for mitigating financial barriers that hinder timely receipt of care.

## Present

We used the Medical Expenditure Panel Survey to assess resource use and expenditures among adult female patients with breast cancer in the USA from 2007 to 2017. Nearly 43% of patients cited financial barriers as the primary reason for D/F care. Family income at 0–125% of the poverty line was a significant predictor of D/F care. Patients reporting D/F care had markedly higher rates of inpatient hospitalization and inpatient expenditures compared with those without D/F care. Notably, annual per capita inpatient expenditures were more than USD $5000 higher for patients with D/F care.^4^ These findings highlight that D/F care, driven in part by inequitable access to medical care, may be a critical contributor to rising healthcare expenditures in the breast cancer population.

## Future

Policy interventions aiming to reduce resource use and expenditures among patients with breast cancer may benefit from focusing on improving access to timely care. Further work is needed to identify and address the root causes of D/F care, particularly financial barriers that disproportionately affect low-income populations. Expanding insurance coverage, reducing out-of-pocket costs, and addressing nonfinancial barriers (e.g., access to transportation and childcare) are important interventions for policymakers to consider. Longitudinal studies are needed to assess the impact of timely receipt of care on resource use and expenditures over longer time horizons.

## References

[1] National Cancer Institute. Financial burden of cancer care. https://progressreport.cancer.gov/after/economic_burden. Accessed 1 Aug 2023.

[2] Lo-Fo-Wong DN, Sitnikova K, Sprangers MA, de Haes HC. Predictors of health care use of women with breast cancer: a systematic review. *Breast J*. 2015;21(5):508–13. 10.1111/tbj.12447. 26132228 10.1111/tbj.12447

[3] Sun L, Legood R, Dos-Santos-Silva I, Gaiha SM, Sadique Z. Global treatment costs of breast cancer by stage: a systematic review. *PLoS ONE*. 2018;13(11):e0207993. 10.1371/journal.pone.0207993. 30475890 10.1371/journal.pone.0207993 PMC6258130

[4] Reddy KP, Jarrell K, Berkowitz C, et al. Association between delayed/forgone medical care and resource utilization among women with breast cancer in the United States. *Ann Surg Oncol*. 2024. 10.1245/s10434-024-16586-x. 39694997 10.1245/s10434-024-16586-x PMC11882630

